# Bleeding gastric cancer in young and elderly patients

**Published:** 2015

**Authors:** X Pucheanu, M Beuran

**Affiliations:** * Dr. Caius Tiberiu Spârchez City Hospital, Zarnesti, Brasov, Romania; ** Department of Surgery, Emergency Hospital Bucharest, Romania

**Keywords:** bleeding gastric cancer, young people, elderly, gastric adenocarcinomas

## Abstract

**Purpose:** This study tried to find the differences between gastric cancer in young and elderly, in addition through the importance of the presence of the upper gastrointestinal bleeding, with two examples of clinical cases.

**Methods:** Two groups of patients divided by age were compared. The first group consisted of 13 cases of patients aged between 32 and 41, and the second consisted of 15 cases, aged 80 to 87 years. The variables considered were: sex, personal history and family history, onset-admission interval, number of days of hospitalization after surgery, the number of days until discharge, personal history/ family history, tumor location, admission diagnosis, intervention type, value hemoglobin on admission, the way externalizing hemorrhage appeared, stage, tumor type/ degree of differentiation of its kind lymph dissection, postoperative complications and deaths.

**Results:** The interval from symptom onset to hospital admission was higher in young people with a greater weight loss and malignant ulcer history or upload family were smokers, but apparently with a lower complication rate. In the elderly, the anemic syndrome was the main event and the complications were more related to comorbidities.

**Conclusions:** Prolonged gastric distress in young patients, associated with smoking, personal history of ulcer and family history of neoplasia should guide the diagnosis to gastric cancer. Anemic syndrome in the elderly may be due to the gastric cancer, and complications are due to comorbidities.

## Introduction

Gastric cancer is the second cause of death in Romania after colorectal cancer, with a total of 650,000 deaths per year worldwide [**1**,**3**]. The highest incidence is reported in Japan, China, Korea, Chile, Colombia, Venezuela, Iceland [**2**].

In Romania, gastric cancer incidence decreased to 73.3% in 1960 from 73.4% in men and in women in 1980 to 56.2%, 48.4% respectively [**2**].

In 2012, the European Cancer Observatory for our country estimated the incidence of gastric cancer in men on the 6th and 8th place in women, with a male: female 2: 1 [**4**].

The factors that the prognosis of patients with gastric cancer depends on are related to the patient and tumor gastric course of treatment [**5**].

Hospital admission of a patient with a complication of gastric cancer appears to cause a reduction in survival at 6 months compared to 12 months for patients without complications [**4**].

In the following study, we tried to see if there are important factors that are significant for young and older patients.

## Materials and methods

We studied two groups of patients with gastric cancer who were operated in the Emergency Hospital Bucharest between 2005 and 2009. Group 1 consisted of 13 young patients in which we included patients aged between 32 and 41 years, and in group 2, patients aged 80 to 87 years (15 patients).

**Chart 1:**
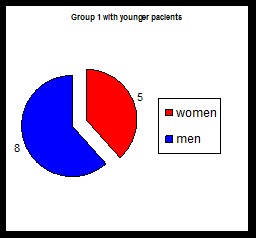
Number of cases in group 1 in younger patients

**Chart 2:**
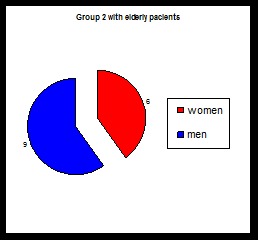
Number of cases in group 2 in elderly

Number of days from stroke onset to hospitalization ranged from 3 months to 1 year in group 1 and between 1 month and 5 months for patients in group 2.

**Table 1:** Range symptom onset - admission

Interval debut - admission	
Group 1 - youth	Group 2 - elderly
3 months – 1 year	1 month – 5 months
Average value 7,75 months	Average value 2,6 months

The number of days of hospitalization after surgery were between 0 and 6 days for patients in group 1 (mean 1.6 days) and between 0 and 7 days for patients of group 2 (with an average of 1.78 days).

Number of days until discharge from hospital patients was as follows:

**Table 2:** Number of days until patient discharge from hospital

Number of days after surgery until discharge	
Group 1	Group 2
9 – 35 days	3 – 21 days
Average value 9,71 days	Average value 13 days

**Table 3:** Personal history;A history of the patient:

	Group 1	Group 2
Weight decreased	5 patients (38,46 %)	3 patients (20 %)
History of ulcers	3 patients (23,07 %)	1 patient (6,66 %) !!!!
History of cardiology	-	5 patients (40 %)
Smoking	6 patients (46,15 %)	-
Medication	Cardiology – 1 patients For ulcer – 4 patients	Cardiology – 4 patients

Family history revealed a malignant load for lot 1 in 3 patients as it follows: 1 patient with gastric tumor - mother, 1 patient with pulmonary tumor - father, 1 patient with hepatic tumor grandmother. For patients in group 2, 2 of them were diagnosed with heart disease/ hypertension.

The diagnosis for the 2 groups was the following:

• Lot 1:

o gastric tumor - 6 cases

o pyloric stenosis - 4 cases

o gastric ulcer - 1 case

o gastric lymphoma - 1 case

o a mechanical jaundice (pancreatic head tumor) - 1 case

• Lot 2:

o gastric cancer - 13 cases

o anemic syndrome - 1 case

o generalized peritonitis - 1 case

Tumor location was on the upper, middle or lower side, and diffused in the stomach as follows:

**Chart 3:**
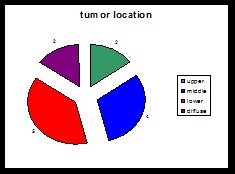
Location of tumor in group 1

**Chart 4:**
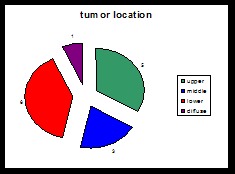
Location of tumor in group 2

On admission, there were patients in both groups who were hospitalized for upper gastrointestinal bleeding (HDS) externalized through haematemesis, melaena, occult or not obvious HDS. There was a case with HDS externalized by haematemesis in group 1 and 3 patients with occult bleeding. There was 1 case of haematemesis and melaena manifested HDS in group 2, only one melena and 6 cases of occult bleeding.

Hemoglobin on admission on the 2 groups was the following:

**Table 4:** The amount of hemoglobin on admission in the 2 groups

	Group 1	Group 2
range	7,3 – over 12 g/ dl	7,4 – over 12 g/ dl
7,3 – 8	1 case	2 cases
8,1 – 10	3 cases	3 cases
10,1 – 12	1 case	3 cases
More than 12 g/ dl	8 cases	7 cases

The types of surgeries performed in the 2 groups were the following:

• Lot 1:

o an exploratory laparotomy - 2 cases

o a Gastroenteroanastomosis - 1 case

o an upper pole Esogastrectomy with anastomosis esogastric 2 cases

o a subtotal gastrectomy - 3 cases (2 gastrojejunal anastomosis on the Roux-Y loop and 1 gastroduodenal anastomosis type Pean)

o a total gastrectomy - 5 cases (2 esojejunal anastomosis on the Roux-Y loop, 2 with loop interposed type Henley and 1 in omega loop with fistula Braun)

• Lot 2:

o a tumor ablation of gastric wall - 2 cases

o a Gastroraphy - 1 case

o an exploratory laparotomy - 2 cases

o an upper pole Esogastrectomy - 1 case 

o a subtotal gastrectomy with gastroduodenal anastomosis type Pean - 4 cases 

o a total gastrectomy - 5 cases (three with esojejunal anastomosis on the Roux-Y loop, 1 with interposed loop type Henley and 1 in omega loop with fistula Braun)

Expanding lymphadenectomy was assessed in group 1 type D1 and D2 in 7 cases and in 3 cases, and in group 2, D2 and D1 5 cases in 5 cases.

The tumor stage was the following (according with UICC/ AJCC and Japanese Classification of Gastric Cancer – [**9**,**10**]):

**Table 5:** Staging cases in the 2 groups

Stage	Group 1	Group 2
II	1	1
IIIB	2	2
IV	10	10
There was no case	-	2

The operated tumors were gastric adenocarcinomas with different degrees of differentiation. For tumors in group 1, there were well differentiated tumors It G1 - 4 cases, moderately differentiated G2 - 2 cases and poorly differentiated G3 - 7 cases. For tumors in group 2 the degree of differentiation was the following: well differentiated G1 - 3 cases, moderately differentiated G2 - 8 cases, undifferentiated G4 - 2 cases.

Complications of 2 groups:

• Lot 1:

o one left subphrenic abscess that required re-intervention for evacuation

• Lot 2:

o one case of fistula of jejunoduodenal anastomosis

o one case of pleurisy

o one case of early postoperative acute pulmonary edema

## Results and discussion

The 2 groups studied were similar in a number of patients and their sex ratio, respecting what we have found in the literature. A study showed that women in Jordan are predominant among the group of younger patients [**6**].

Regarding the interval from the symptom onset, we found out that it was longer for younger patients. This may indicate that young people interpret the symptoms as an ulcer, moreover the gastric antisecretory medication take a longer period of time and prolong the time to professional medical consult [**7**].

The interval from admission to surgery was similar in both groups.

Dates until patient discharge from hospital were higher on average in the group of elderly patients and it seems to relate to the comorbidities they have.

From the personal history of patients under 40 years we have seen that a larger number of these patients on admission suffered a significant weight loss, even 20 kg, had a history of ulcers, were smokers, but no other comorbidities as such were those of group 2 (cardiology and related medication history). Regarding the family history, we revealed a malignant upload more important to patients in group 1. This may be partially correct given that the patients in group 2 may have incomplete information about their predecessors related to a diagnosis of death [**6**].

The diagnosis was gastric tumor in a higher percentage in group 2 (86.66%), those in group 1 having the admission diagnosis of ulcer with pyloric stenosis or stenosis or obstructive jaundice [**15**].

For tumor location, we did not find significant differences between the 2 groups.

Given that in group 1, the studied cases were urgently hospitalized, we evaluated the number of days of hospitalization and before surgery. We found that only a small number of cases were operated immediately after admission, which means that upper gastrointestinal bleeding can be mastered by endoscopic means providing time to balance patient and prepare it for safe operation [**6**].

Regarding tumor location, we found that most of the cases were tumors of the middle and lower portion of the stomach, as recorded in literature.

Hemoglobin level at admission was similar in the 2 groups although the externalization modality was occult in more cases in group 2 (6 cases, i.e. 40%).

Surgery consisted of exploratory laparotomy, subtotal or total gastrectomy, and the removal of a tumor stromal and tumor gastrorrhaphy perforated and bleeding, surgical resources above, the analogy for both groups. The surgical resection was associated D1 or D2 lymphadenectomy type. Type D2 lymphadenectomy was performed in about 53.84% of the patients in group 1, whereas for patients in group 2 of the predominant D1 (40%) in 33.33% D 2 being cut therefrom [**7**,**11**,**13**].

Staging cases for both groups was predominantly stage IV, 76.92% in group 1 and 66.66% respectively in group 2. Similar results have been presented in 2013 in a study published by Mexican authors [**7**].

Complications of 2 lots. In group 1 it was about one subphrenic abscess left, which required reoperation for drainage (it can be found detailed below). In group 2 there were 3 complications: early postoperative acute pulmonary edema that required intensive care and has evolved favorably, a significant pleural effusion requiring thoracentesis and jejunoduodenal anastomosis fistula after total gastrectomy with interposed (Henley) jejunal loop. The evolution of these three cases was favorable in immediate postoperative patients discharged within 21 days after surgery [**12**].

Postoperative complications were more frequent in the group of elderly patients mainly because of comorbidities [**8**].

Beyond that, curative gastrectomy with lymph node dissection should have been performed in very old gastric cancer patients [**14**].

***Presentation Case 1:***

A patient aged 33, from urban area, was met with gastric ulcer and refractory to treatment with proton pump inhibitors for 4 months, smoker, was hospitalized for about 8 kg weight loss in the last 4 months and pain in the epigastrium. From the personal history, we remembered paternal grandmother with liver cancer. On examination, the abdomen was painful in epigastrium, with no signs of peritoneal irritation.

Picture biological admission: HGB 7.4 g/ dl, CA 19-9 and CEA normal.

Radioscopy empty pulmonary and abdominal ultrasound revealed no pathological lesions.

Upper gastrointestinal endoscopy showed an ulcerovegetant tumor, 5/ 6 cm, the greater curvature, which extended to the back, anfractuous edges, deep crater with fibrin clots. Biopsy was positive for adenocarcinoma.

Abdominal CT within normal limits!?

The surgery was performed and revealed a rear-sided gastric tumor and a large curvature, with transverse mesocolon invasion. Intraperitoneal cytology was positive for atypical. The total gastrectomy with eso-jejuno-anastomosis practice with loop interposed Henley and segmental colectomy.

Tumor stage was pT4N2Mx (per) R0D2.

Histopathology: moderately differentiated gastric adenocarcinoma with signet ring cells.

Evolution was encumbered by the appearance of a left subphrenic abscess after a very small flow fistula that required re-intervention for drainage, later evolving favorably.

The importance of this event:

• Young patient, smoker, known gastric ulcer treated with proton pump inhibitors

• family history of liver cancer paternal grandmother notes

• On admission moderate chronic anemic syndrome

• Endoscopy revealed a giant gastric ulcer high curvature, biopsy positive for adenocarcinoma

• intraperitoneal fluid cytology atypia highlights

• The surgical intervention consisted of a total gastrectomy with jejunal loop interposed Henley, with segmental colectomy

• In terms of histopathology, it was a moderately differentiated adenocarcinoma with signet ring cells, aggressive form, according to which we found in literature they are specific to young patients and correlate with bad prognosis [**7**]

• Evolution was favorable and the patient discharged 35 days after surgery

• Case was significant for a young patient with aggressive histopathological form.

***Presentation Case 2:***

A patient aged 82, from rural area, reported the presence of epigastric pain with nausea, vomiting, loss of appetite, meat food intolerance, weight loss of about 6 kg in 2 months, satiety early.

From family history of heart uploading, we remembered that all family members had hypertension.

The personal history revealed hypertension, prostate adenoma, cataract.

The admission biological picture: HGB 10.1 g/ dl.

Abdominal CT: bulky tumor formation in the antropyloric region, 8 cm, with significant infiltration of the gastric wall, luminal stenosis secondary lymph nodes of 5-6 mm on the lesser curvature.

The surgical intervention and subtotal gastrectomy practice Pean gastroduodenal anastomosis.

Tumor stage was pT3N1M0.

Intraoperative cytology is negative for showing atypical reactive mesothelial cells.

Histopathological examination: poorly differentiated adenocarcinoma infiltrating the gastric wall completely and adipose tissue adjacent nodes in Group 4 (2 of 3) bulky metastatic adenocarcinoma; remaining nodes examined the reagents (were basically positive for neoplasia 2 of 17).

Favorable postoperative course of the patient was immediately postoperatively.

Consultation oncologic surgery specialist treatment was not recommended.

The patient is alive 9 years after this episode, upper gastrointestinal endoscopy and abdominal CT control to 8 years not showing signs of local recurrence or distant gastric tumor.

The case was chosen for the following aspects:

•Elderly patients without a family history of cancer, without a personal history of suffering gastric easy anemic syndrome

•Investigations revealed a gastric antral tumor and perigastric lymph 

•The practice subtotal gastrectomy intraoperative gastroduodenal anastomosis type D2 lymphadenectomy type Pean and

•postoperative course was simple

•In terms of histopathology it was a poorly differentiated adenocarcinoma with multiple metastases in the peri-regional lymph (4 of 20)

•The patient is alive 9 years after the intervention, without suffering consecutive digestive gastric surgery.

## Conclusions

1. in general and in particular, gastric cancer represents a challenge for the surgeon

2. a young patient with gastric cancer had a history of prolonged gastric distress, smoking, weight loss more than the elderly, and, in terms of location, pyloric tumors were located in the mediogastric region. Prolonged gastric suffering under fair treatment should make us think about gastric cancer in young patients as well

3. in elderly patients, the manifestation of gastric cancer can be similar to the anemic syndrome. Tumors are located in all parts of the stomach, and the degree of differentiation is the following: there are well differentiated tumors (G1 or G2) than in young patients in whom they are poorly differentiated (G3)

4. upper gastrointestinal bleeding regardless of the externalizing, does not appear to be an important factor in the evolution of the extremities patients ages

5. postoperative complications are more in the group of elderly patients mainly because of comorbidities

6. the present study is retrospective also on small batches and requires prospective randomized re-evaluation 

7. regarding resection and lymphadenectomy, pre and postoperative care, there are no differences between the 2 groups, showing that age does not dictate surgery

8. it is important to realize a randomized prospective study on larger groups of patients to be statistically significant.

**Disclaimer**

Patients in the study are part of a larger group of patients with gastric cancer surgery in the Emergency Hospital between 2005 and 2009, half of whom suffer from upper gastrointestinal bleeding.

## References

[1] Ian M, McKinley A (1998). Ordinul nr. 1216/ 2010 privind aprobarea Ghidurilor de practică medicală pentru specialitatea gastroenterologie, Cancerul gastric. www.ms.ro.

[2] Andronic D, Georgescu SO (2003). Cancerul gastric.

[3] Fuchs CS, Mayer RJ (1995). Gastric carcinoma. N Engl J Med.

[4] Grigorescu M, Irimie A, Beuran M (2013). Tratat de oncologie digestivă, vol I, Cancerul esofagian şi gastric.

[5] Marrelli D, Caruso S, Roviello F (2012). Prognostic factors and score. Systems in gastric cancer, in Manzoni G, Roviello F, Siquini W. Surgery in the multimodal management of gastric cancer.

[6] Bani-Hani KE (2005). Clinicopathological comparison between young and old patients with gastric adenocarcinoma. Int J Gastrointest Cancer.

[7] Lopez-Basave HN, Morales Vasquez F, Ruiz-Molina JM, Namendys-Silva SA, Vela-Sarmiento I, Ruan JM, Rosciano AEP (2013). Gastric cancer in young people under 30 years of age: worse prognosis, or delay in diagnosis?. Cancer Manag Ress.

[8] Pisanu A, Montisci A, Piu S, Uccheddu A (2007). Curative surgery for gastric cancer in the elderly; treatment decision, surgical morbidity, mortality, prognosis and quality of life. Tumori.

[9] (2010). AJCC: Cancer Staging Manual.

[10] (1998). Japanese Classification of Gastric Carcinoma - 2nd English Edition - Gastric Cancer.

[11] Vasilescu C, Trandafir B (2011). Probleme de chirurgie oncologicã 2. Lecţia japonezã. Limfadenectomia D2 în cancerul gastric. Chirurgia.

[12] Saif MW, Makrilia N, Zalonis A, Merikias M, Syrigos K (2010). Gastric cancer in the elderly: an overwiew. www.ejso.com.

[13] Katai H, Sasako M, Sano T, Maruyama K (1998). The outcome of surgical treatment for gastric carcinoma in the eldrely. Japanese Journal of Clinical Oncology.

[14] Ryol Lee S, Ook Kim H, Hak Yoo C (2012). Impact of chronologic age in the elderly with gastric cancer. J Korean Surg Soc.

[15] Santoro R, Carboni F, Lepiane P, Ettorre GM, Santoro E (2007). Clinicopathological features and prognosis of gastric cancer in young European adults.

